# Molecular identification of papillomavirus in ducks

**DOI:** 10.1038/s41598-018-27373-6

**Published:** 2018-06-14

**Authors:** Richard A. J. Williams, Conny Tolf, Jonas Waldenström

**Affiliations:** Center for Ecology and Evolution in Microbial Model Systems (EEMiS), Linnaeus University, Kalmar, Sweden

## Abstract

Papillomaviruses infect many vertebrates, including birds. Persistent infections by some strains can cause malignant proliferation of cells (i.e. cancer), though more typically infections cause benign tumours, or may be completely subclinical. Sometimes extensive, persistent tumours are recorded–notably in chaffinches and humans. In 2016, a novel papillomavirus genotype was characterized from a duck faecal microbiome, in Bhopal, India; the sixth papillomavirus genotype from birds. Prompted by this finding, we screened 160 cloacal swabs and 968 faecal samples collected from 299 ducks sampled at Ottenby Bird Observatory, Sweden in 2015, using a newly designed real-time PCR. Twenty one samples (1.9%) from six individuals (2%) were positive. Eighteen sequences were identical to the published genotype, duck papillomavirus 1. One additional novel genotype was recovered from three samples. Both genotypes were recovered from a wild strain domestic mallard that was infected for more than 60 days with each genotype. All positive individuals were adult (P = 0.004). Significantly more positive samples were detected from swabs than faecal samples (P < 0.0001). Sample type data suggests transmission may be via direct contact, and only infrequently, via the oral-faecal route. Infection in only adult birds supports the hypothesis that this virus is sexually transmitted, though more work is required to verify this.

## Introduction

Papillomaviruses (PV) have circular dsDNA genomes of ~8 kb usually encoding seven or eight conserved proteins^1^. They exhibit strict tropism for squamous and mucosal epithelium, and are generally considered to show high specificity to host species^2^. They frequently manifest as easily identified wart-like growths of the skin, or more easily overlooked infections of internal epithelia. Infections usually range from generally mild, often subclinical infections, to *benign* tumours, papillomas, or warts. However, a number of PVs are pathogens associated with or causing malignant transformation (*i*.*e*. the process by which cells become cancerous). Most infamously, human papillomavirus (HPV) DNA is found in nearly 100% of cervical cancer^3^, and about 80% of *Sylvilagus* rabbits lab-infected with Shope papillomavirus (also known as *Sylvilagus floridanus* papillomavirus, SfPV) developed malignant cutaneous tumours^4^. HPV is probably the most common sexually transmitted infection, though most cases (90%) regress within two years, and few lead to invasive cancer^5^. At least 30 HPV types have been shown to cause cervical cancer^6^, and mucosal HPV types cause other genital, head and neck cancers^7^. It is further hypothesized that Beta HPV types play a role in non-melanoma skin cancer development^7^. PV also causes cancer in cattle, dogs, and horses^8^. Infrequently, PV infections lead to extensive wart and tumour growths, which have a high risk of developing into skin cancer, such as *Epidermodysplasia verruciformis*^9^, or SfPV^10^.

The virus family contains 353 known PV types (the Papillomavirus Episteme (PaVE) available at https://pave.niaid.nih.gov/^11^), defined as complete PV genomes that have been cloned and for which the L1 ORF sequences are more than 10% different from the most similar known taxa^12^. Most PV types are known from humans (>183) and other mammals (>160). Domestic mammals, such as cattle and dogs, are particularly well represented with 23 Bovine PV types and 20 Canine PV types, respectively (PaVE; available at https://pave.niaid.nih.gov/^11^). Only eight genotypes have been found in birds^13–15^—from diverse avian orders—three from reptiles^16^ and one from a fish^17^. Recently, a new PV genotype was described from a duck microbiome collected in Bhopal, India. This virus, designated as duck PV (hereafter duck PV1), was detected as three sequences of about 7500 bp in total, which is approximately the length of a full PV genome^18^.

It is striking that only eight PV genotypes have been found amongst the approximately 10,000 species in Class Aves^19^, and that only one avian PV host species has been found to host more than one PV genotype^20^. This imbalance between mammal and other taxa suggests that humans and closely associated domestic and zoo mammals, are better studied than other hosts, so that the diversity of PVs in non-mammal (and non-human) hosts is vastly underestimated^21^. Alternatively, there may be genuine evolutionary and ecological reasons for the high diversity in some hosts; some viral agents seem to have infected *Homo sapiens* from early in our evolutionary history and have a deep-rooted relationship with human ancestors^22^.

The currently known avian papilloma types are highly divergent with less than 65% genomic identity^14,15,23^ and form an avian-reptilian clade^24^, which appears to include the single PV detected in a fish^17^. Little is known about the ecology of avian PVs. Three cause large, obvious cutaneous tumours: FcPV1 in chaffinch (*Fringilla coelebs*)^25^, PePV1 in African grey parrot (*Psittacus erithacus*)^26^, FgPV1 in northern fulmar (*Fulmarus glacialis*)^14^. FlPV1 was recovered from a cutaneous swab of an apparently normal leg in yellow-necked francolin (*Francolinus leucoscepus*)^27^, PaPV1 from a faecal sample^23^, and PaPV2 from a cloacal swab^20^, in Adélie penguin (*Pygoscelis adeliae*), ScPV1 from the apparently normal tongue of a dead captive canary (*Serinus canaria* domestica)^15^, and lastly, duck PV, from a metagenomic analysis of a duck gut (*Anas platyrhynchos* domestica)^18^. The best known avian PV, FcPV1, has been observed frequently by ornithologists^28^, and was even recovered from a 1930s museum specimen^29^. One large scale study in Holland (*N* = c.25000) determined a prevalence of chaffinch leg lesions of 1.3%^30^—though diagnosis was generally by visual inspection.

Recently the recovery rate of novel PV genomes has increased, in both human and animal sources^11^. This has been driven by the advent of new molecular tools such as w29 DNA polymerase used in rolling-circle amplification and next-generation sequencing (NGS)^21^, and has greatly improved our understanding of the diversity of PV. Duck PV was detected from one such metagenomic screening of the duck gut microbiome^18^. Domestic ducks are an important livestock animal; with nearly 5 billion domestic ducks, they are the second most numerous poultry animal after chickens^31^. The wild ancestor of most domestic ducks, the mallard (*Anas platyrhynchos*) is very common (>19 million individuals)^32^, migratory, and an important host for avian influenza virus^33^.

The discovery of a new PV, with unknown epidemiology, prompted us to screen 1128 cloacal swabs and faecal samples collected from 299 ducks sampled at Ottenby Bird Observatory, Sweden in 2015, using a newly designed real-time PCR. We tested whether screening a large panel of individual mallards revealed the presence of duck PV, or if the first detection was an infrequent occurrence. In the former case, we aimed to establish prevalence, detect infection patterns, and, if possible, assess genetic variation in duck PV lineages.

## Results

We designed a novel real-time PCR that was able to detect duck papillomavirus quickly and efficiently. The DNA concentration of the undiluted positive control was 37.7 ng/µl. We detected pure positive control at dilutions of up to 10E-8 (estimated as 437 copies), which was, as anticipated, more sensitive than using traditional PCR alone (where amplicons of positive control produced a band of the anticipated size at dilutions of only 10 E-7). In a direct challenge between PCR and real-time PCR, only 14/21 (66.6%) of real-time PCR positive samples tested positive for papillomavirus following PCR with bands of the anticipated size. In all cases, samples that could not be amplified using direct PCR were those that produced the weakest signal in the real-time PCR (higher Ct values, weaker fluorescent signal at the melting peak, and often containing peaks associated with primer dimers). The Ct values for real-time PCR positive samples that were amplified by traditional PCR (with Amplitaq) averaged 25.1 compared to average Ct values of 29.1 for those that did not. All of the real-time positive samples that had tested negative using traditional PCR were further challenged using PlatinumTaq Polymerase (ThermoFisher Scientific). Using this high fidelity polymerase, these samples then produced good quality bands and sequences. The sequences from ten samples (four individuals; five amplified using Amplitaq, and five using PlatinumTaq) were occasionally noisy, particularly on the forward primer. This raises the question of whether more than one sequence was competing for amplification – if so the sequence or sequences were either much less common in the sample, or possibly more variable at primer hybridization sites.

We detected papillomavirus DNA in 21/1128 samples (1.9%) and in 6/299 individuals (2%). The prevalence was higher in lure ducks (farm reared female mallards used to attract wild ducks) (2/17: 11.8%) compared to wild mallards (4/246; 1.6%); all Eurasian teal (*Anas crecca*; N = 35) and the single Eurasian wigeon (*Anas penelope*) were negative (Table 1), though the number tested was also low. One individual, lure duck 1, was positive 14/38 times she was tested. Two wild individuals were positive 2/16 times and 2/2 times respectively. One lure duck and two additional wild mallards were positive once (Table 2).

**Table 1:** Summary of the number of samples and individuals tested and positive (Pos) in this study, and the papillomavirus prevalence (Prev) detected in each group.

Species name	Common name	# of samples	Pos	Prev (%)	# of individuals	Pos	Prev (%)
*Anas crecca*	Eurasian Teal	35	0	0	35	0	0
*A*. *penelope*	Eurasian Wigeon	1	0	0	1	0	0
*A*. *platyrhynchos*	Mallard	586	6	1.0	246	4	1.6
*A*. *platyrhynchos* domestica	Lure ducks	506	15	3.0	17	2	11.8
Total		1128	21	1.9	299	6	2

**Table 2:** Summary of sample types (faecal and cloacal swab) from individuals that tested positive in this study, showing the number that were duck papillomavirus (PV) positive (Pos), the number that were tested and the % of samples that tested duck PV positive (Pos %).

Subject name	Unique identifier	Faecal Pos	Faecal test	Faecal Pos %	Swab Pos	Swab test	Swab Pos %
Lure duck 1	90A87175	1	19	5.26	13	19	68.42
Lure duck 2	90A87174	0	26	0.00	1	12	8.33
Mallard 1	90A93486	0	13	0.00	2	3	66.67
Mallard 2	90B12558	1	4	25.00	0	0	0.00
Mallard 3	90B12811	1	1	100.00	0	0	0.00
Mallard 4	90B13192	1	1	100.00	1	1	100.00
Total		4	64	6.25	17	35	48.57

Seventeen of one hundred and sixty swab samples were duck PV positive (10.6%; Table 1), which was significantly higher (Yates corrected Chi Square = 72.9; P < 0.0001) than the number of positive faecal samples (4/968; 0.4%). Four of the thirty-seven individuals (10.8%) subjected to cloacal swabbing, were positive, compared to 4/298 individuals (1.3%) whose faecal samples were tested (*N* = 299; two-tailed Fisher test; P = 0.0064). Seventeen of thirty-five swabs (48.6%) from positive individuals were positive, compared to only 4/64 (6.25%) positive faecal samples (not significant). Lure duck 1 was particularly revealing in this regard. Thirteen of nineteen cloacal swabs tested positive compared to only 1/20 faecal samples. Similarly, Mallard 1 faecal samples tested negative 13/13 times, whereas cloacal swab samples were positive 2/3 times. The one positive sample from lure duck 2 was also from a cloacal swab, with 11 and 26 cloacal swab and faecal samples negative, respectively. The positive samples from the other mallards were mainly from faecal samples (3/4), and no cloacal swab samples tested negative in these individuals. All positive samples were found in adults (6/96), while the 150 first year birds were negative (*N* = 246; two-tailed Fisher test; P = 0.004).

**Figure 1:**
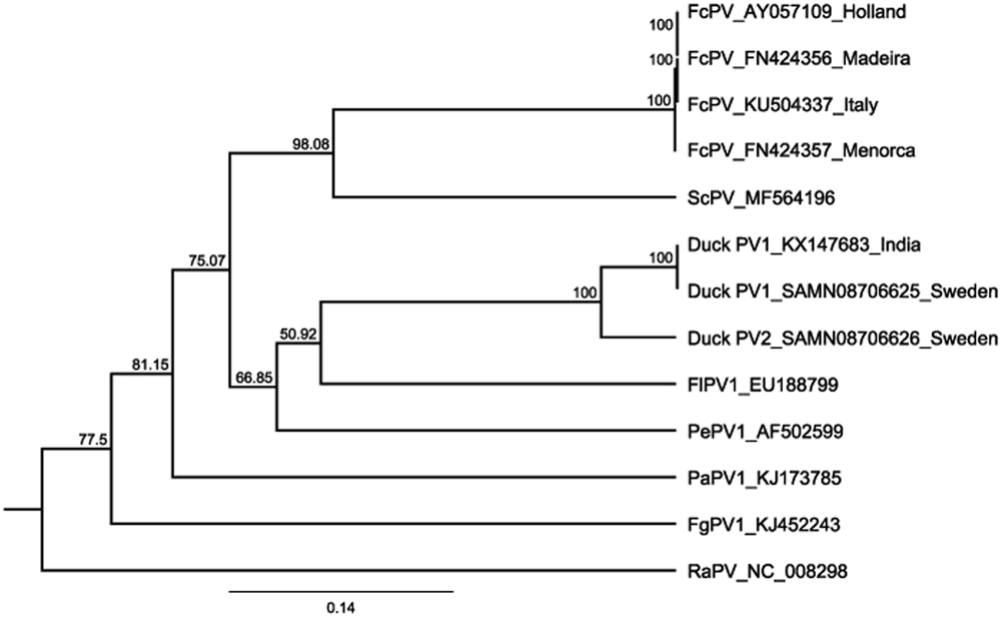
UPMGA guide tree constructed using the 394 nucleotide sequence for the BCon region of the L1 gene for all published avian papillomavirus sequences, with the exception of PaPV2. *Rousettus aegyptiacus* papillomavirus 1 (RAPV1) is used as outgroup. Bootstrap values are shown in bold, and the scale bar represents the number of amino acid differences per site.

We were able to amplify duck BCon sequences (of 394 bp) from all real-time positive samples. Eighteen of twenty one sequences, hereafter referred to as duck PV1, were identical to the published genotype (Genbank Acc No: KX147683). This sequence was found in all positive individuals. In addition, three identical sequences, hereafter referred to as duck PV2, that shared about 91% nucleotide sequence identity to the published genotype (Genbank Acc No: KX147683), were also found in one lure duck. The amino acid sequence of this second genotype was 98% identical to duck PV1 amino acid sequence ANN29877 (129/132 AA identity). By contrast the difference to this amino acid sequence and the other avian papillomavirus genotypes varied from 60–75%. Possibly this real-time PCR was less sensitive in the detection of the novel strain. Two of the three sequences for the novel genotype proved difficult to amplify and sequence, compared to six of eighteen of the previously known sequence (from which the primers were designed), though this difference was not significant. Phylogenetic analyses revealed that duck PV 1 and duck PV2 are most closely related to one another, and form a sister clade to FlPV1 (Fig. 1).

Both viruses appeared to cause long-lasting infection in lure duck 1. Duck PV1 was first detected in lure duck 1 on 9^th^ September, and last on 4^th^ December, a total of 84 days. Two positive wild ducks were duck PV1 positive for at least 27 and 17 days. Duck PV2 was detected in lure duck 2 on 8^th^ October, and again on the 26^th^ and 27^th^ November, a total of 60 days. No clear cycle emerged for the occurrence of either PV genotype in lure duck 1, though data are few. Lure duck 1 was positive 14/38 times tested. It was positive 8/18 times it was sampled between the 7^th^ October and 29^th^ November. This consisted of nine cloacal swabs and nine faecal samples. Five of these samples (four cloacal swabs, one faecal sample) were positive for duck PV1, and three (all cloacal swabs) were positive for duck PV2. Two swab samples were negative. Duck PV1 was detected one and two days before the detection of duck PV2, and two and three days after. Faecal samples from the 9^th^ October and 28^th^ November (the day after the detection of duck PV2), were negative, though faecal samples were apparently a poorer sample type than cloacal swabs. Only one faecal sample was positive, on the 11^th^ October, for duck PV1.

## Discussion

Duck PV is a newly detected virus with unknown epidemiology. Prompted by its detection and gene characterization, we developed a screening assay and tested a large panel of samples collected from both wild and domestic waterfowl, where repeated sampling of individuals across time allows for detecting patterns of infection. We found that this virus was present in both populations, and that the prevalence was higher in lure ducks (11.7%) than in wild mallards (1.6%). These results may contain a false negative rate, however, as the vast majority of samples were faecal and not from the cloacal swabs, with prevalences of 1.3% and 10.8%, respectively, and 5% and 65% for lure duck 1. Mallard 1 also registered a negative test between two positive samples. The low number of lure ducks (*N* = 17), and the high number of repeat samples from lure ducks (e.g. we tested 38 samples from lure duck 1), mean that the datasets are not directly comparable, and differences should be interpreted cautiously.

The only published avian PV prevalence survey, in chaffinches, detected PV in 1.3% of subjects tested^30^, a similar figure to the prevalence in wild mallards. However, this prevalence is only about 10% of the prevalence we detected in cloacal swab samples, which is the more appropriate comparison. The chaffinch figure described the prevalence in individuals with (untested) leg lesions, FcPV1 presence was assumed, not shown, in the vast majority of individuals. Additionally, the chaffinch prevalence figure is based on clinically positive individuals only, while in this survey ducks were tested blind to clinical status. In another study, 12.5% of 40 apparently healthy chaffinches were FcPV positive, which is similar to the prevalence figure found for cloacal swab samples of ducks tested in this study (Williams, R. *Pers Comm*). One review of the prevalence of five HPV types in male genitalia found prevalence varied from 1.35–72.9%^5^. Clearly PV prevalence varies greatly depending on virus type, and other factors. The prevalence we detected in both populations of birds suggests that the virus is widespread and likely underreported (which goes in line with what we know of PV in general).

We detected not only the known duck PV, but also a new strain of duck PV (duck PV 2). Based on the L1 gene fragment, the second genotype is quite different to the first, and may be a new PV species. Both genotypes showed intermittent detection, but patterns suggest that both are persistent infections. Duck PV 1 showed persistence in 3/4 individuals that were sampled multiple times, suggesting that it causes long-lasting or chronic infection. Lure duck 2 tested positive 1/38 times, so presumably the infection did not persist in this individual. Only one of the repeat sampled individuals, Mallard 4, was PV positive every time it was sampled (2/2 times). Papillomavirus DNA was recovered inconsistently from the other two individuals. This is likely, in part, due to the difficulty of recovering PV from faecal samples. However, PV was also recovered inconsistently from cloacal swabs of lure duck 1 and mallard 1 (13/19 and 2/3 times), and two different genotypes were recovered inconsistently from lure duck 1. Alternatively it may reflect poorly understood aspects of the duck PV replication cycle.

Failure to detect virus could be due to sampling material (cloaca vs fecal sample). PVs can be detected in avian faeces (for instance 4/21 positive samples in this study and from PaPV1^23^). Our sampling was heavily biased towards faecal samples (86% of the sample)–owing to the fact that the samples were originally collected as part of an avian influenza monitoring scheme, and that faecal samples yield better results for AI screening^34^ and are more efficient to collect. However, one of our major findings is that cloacal swabs yielded significantly more positive results than faecal samples. This was not surprising as PV is frequently transmitted through direct contact. Many HPV types (>40^35^) are sexually transmitted, and we speculate that this will be the case for some avian PV strains too. In this case, transmission could be direct, faecal, or by another unknown route. All positive birds are adult, which is consistent with direct transmission. This tentative hypothesis merits future exploration.

Greater than 180 Papilloma types have been detected in humans, the species best surveyed for PV, and greater than 20 in both cattle, and dogs. Papillomavirus has been detected in all carefully studied mammals, and also some birds, reptiles and even one fish species. It is rare for a specific papillomavirus to infect two different host species. This means it is highly probable that there are tens of thousands of PVs, or more, that have yet to be described^16^. In contrast to the high diversity of PVs in the best studied mammals, the known PV diversity in birds is low, with PVs identified in only eight species and only one species having two recognised PV genotypes^20^. FcPV, the best studied avian papillomavirus shows virtually no variation in the BCon region of the L1 gene; all but one of the approximately 40 FcPV sequences that have been identified to date are identical (the one exception has one nucleotide substitution, and an identical amino acid sequence). The two PV genotypes recovered from mallard ducks in this study are 91% identical along a 394 bp fragment of the L1 gene.

Several recent papillomavirus studies on related mammalian papillomaviruses have recorded the phenomenon that traditional detection methods (PCR, Rolling Circle Amplification) commonly used for the amplification of papillomavirus genomes failed to uncover the full papillomavirus diversity present in a sample compared to NGS^36,37^. Using NGS avoids the problem of designing broad-spectrum primers capable of amplifying highly variable sequences. NGS can also show whether we have captured the full diversity of papillomavirus species in these positive samples, yield full genomes, which could be used to assess the adequacy of primers used for amplification of papilloma viruses in conventional PCR, as well as for more robust phylogenetic analyses, protein characterization, to assess whether there is any evidence for recombination, etc. On the other hand, other studies have found that papillomaviruses are recovered using PCR but not using NGS^38,39^.

We introduce a new real-time PCR for the rapid detection of at least two strains of papillomavirus in ducks. The more common duck PV is identical to a published sequence and the second has 91% nucleotide sequence identity. Whole genome characterization is required to assess whether it is a new PV type. This study is an important step in describing the ecology of duck PV, and suggests that PV diversity and prevalence is underreported and should prompt more studies. The data at hand suggests duck PV may be sexually transmitted, and can cause persistent infection. Further study is required to assess impacts on duck health, production, and reproductive success.

## Methods

### Sample collection

Wild mallards were captured in a duck trap located at Ottenby Bird Observatory, Sweden (56°12′N, 16°24′E) as part of a long-term avian influenza monitoring scheme^34^. All birds were tagged with a unique identification ring, weighed, and measured. A sample was collected from each individual: either freshly deposited faeces collected from the bottom of a single-use cardboard box, or the cloaca was swabbed for those individuals that did not defecate in the box between capture and processing. Samples were placed in virus transport medium (VTM) and stored at −70 °C within 2–6 h of collection^34^. Additionally, we collected samples from lure ducks on a daily basis throughout the study period. These birds are farm-reared female mallards used to attract wild ducks, and are housed in a compartment within the trap, separated by nylon mesh, but sharing water body with the part of the trap used for capturing wild birds. Sampling was carried out from Julian day 213 to 349 (1 August–15 December 2015). We tested a total of 1128 samples from 299 individuals. The samples were from three wild species, and 17 female mallard lure ducks (Table 1). Lure ducks were raised at a commercial farm for hunting purposes, and were probably exposed to domestic mallard drakes before the sampling. Samples reflected a number of age groups (291 juvenile, 695 adult, 142 unknown age), sexes (688 female, 432 male, seven unknown), and arrival dates across the season. Samples from lure ducks made up 44.7% of our sample. 55% of individuals were only sampled once, and 5.7% of individuals were sampled on more than 10 occasions.

All methods were performed in accordance with the relevant guidelines and regulations according to Swedish law (Svensk författningssamling 2003:1077, https://www.riksdagen.se/sv/dokument-lagar/dokument/svenskforfattningssamling/djurskyddslag-1988534_sfs-1988-534) following an ethical approval from the Swedish Animal Research Ethics Board (“Linköpings djurförsöksetiskanämnd” reference numbers 46-09 and 111-12). This board is delegated by the Swedish Board of Agriculture and oversees that applications adhere to Swedish law.

### Sample screening

Viral DNA was extracted from VTM containing samples with the MagNA Pure 96™ Nucleic Acid Purification System (Roche, Mannheim, Germany) and MagNA Pure 96 DNA and Viral Nucleic Acid Large Volume Kit (Roche) following manufacturer’s recommendations. VTM samples were diluted 1:4 with PBS prior to extraction. Following extraction, samples were assayed by real time PCR using newly designed primers duck-BCon F (5′-TGCCTAAGGTGTCGGCCAACCA-3′) and duck-BCon R (5′-CCAAACCCAATATCACTCAT-3′) designed with reference to the duck papillomavirus L1 gene sequence, targeting the same short (394 bp) BCon region of the avian papillomavirus L1 gene sequence established for other avian papillomavirus types^29^. Briefly, duck PV was screened using a real-time PCR assay with the iQ^™^ SYBR Green Supermix kit (BioRad, Carlsbad, CA). Each reaction consisted of 2 μl DNA template (25 to 50 ng/μl), 5 μl iQ^™^ SYBR Green Supermix, 500 nM each of the forward and reverse primers and DNase free H_2_O to a final volume of 10 μl. Four negative controls comprising extracts from PBS alone and 4 positive controls, in the form of a *de novo* synthesized duck PV L1 gene fragment (Genbank Acc No: KX147683) were included in every 384 plate. The positive control gene fragment, synthesised and cloned into a pUC57 cloning vector by the manufacturer (GenScript, New Jersey, USA), was amplified using the vector specific M13 forward and reverse primers and purified using the Wizard® SV Gel and PCR Clean-Up System (Promega, Madison, WI, USA). Positive control template DNA concentration was measured using a Nanodrop ND 2000 spectrophotometer. Approximate DNA copy number was estimated using an online calculator (http://cels.uri.edu/gsc/cndna.html). Real-time PCR analyses were run on a LightCycler480 with the following thermal cycle: an initial denaturation of 95 °C for three minutes, followed by 45 cycles of 95 °C for 15 s with a ramp rate of 4.8 °C/s, 55 °C for 30 s with a ramp rate of 2.5 °C/s, and 60 °C for 45 s with a ramp rate of 4.8 °C/s, with data acquisition at the end of each elongation step. Immediately following the real-time PCR, a melting curve analysis was performed (95 °C for 15 s with a ramp rate of 4.8 °C/s, 55 °C for one min with a ramp rate of 2.5 °C/s, followed by a slow incremental increase in temperature to 95 °C with a ramp rate of 0.11 °C/s and continuous measurement of fluorescence intensity), finally samples were cooled to 40 °C for 30 s. A threshold cut off (Ct) of 32 was used for all rRT-PCR screens, though samples were only considered positive if they also showed a melting peak at 89C.

### Virus characterization

All samples positive for avian papillomavirus in the real-time PCR screening were amplified for sequencing using a conventional PCR with the same duck-BCon primers resulting in a 394 bp amplicon. The reaction contained 2.5 µl of 10× Buffer, 1.5 mM of additional MgCl_2_, 0.2 mM of each dNTP, 500 nM of each primer, 0.1 units AmpliTaq Polymerase (Promega, Fitchburg, WI, USA), 2 μl DNA template (25 to 50 ng), and water to a final volume of 25 μl. Thermocycling conditions were 94 °C for 10 min, followed by 40 cycles of 94 °C for 30 s, 50 °C for 30 s, 72 °C for 1 min, followed by a final extension at 72 °C for 10 min. Amplified DNA was visualised by loading 3 μl of PCR product in 1.5% agarose gels stained with SYBR Safe DNA Gel Stain (ThermoFisher Scientific, Waltham, MA, USA). The PCR products were cleaned using the Wizard® SV Gel and PCR Clean-Up System (Promega, Madison, WI, USA) according to manufacturer’s instructions. Eight real-time positive samples that could not be amplified, and two that yielded poor sequences, using the above PCR protocol, were retested using the same PCR protocol with the substitution of PlatinumTaq Polymerase (ThermoFisher Scientific). PCR products were sequenced with the duck-BCon primers at Macrogen Europe (Amsterdam, Netherlands). Sequences were confirmed by Sanger sequencing from both ends using both the duck-BCon forward and the reverse primers.

### Phylogenetic Analyses

Nucleotide sequences were analysed using Geneious version 7.1.9 (Biomatters, New Zealand). The 394 bp consensus sequences were compared to published sequences using the NCBI Blast software (https://blast.ncbi.nlm.nih.gov/blast/Blast.cgi), but excluding the BCon region from PaPV2 (MF168943), as PaPV2 is highly divergent in this region, and the topology recovered was unrealistic compared to full genomic analysis. Sequences were assembled and subsequently aligned with the MAFFT algorithm^40^ in Geneious version 7.1.9, and including a homologous sequence of *Rousettus aegyptiacus* papillomavirus 1 as outgroup. Phylogenetic models were determined and UPMGA guide trees were built and bootstrapped 10000 times. All sequences have been deposited in GenBank (Accession nos SAMN08706620-SAMN08706626).

### Statistical Testing

Yates corrected Chi Square tests and Fisher’s exact tests, when sample size was lower than 1000, were performed using Statistica 7 (Dell, Round Rock, TX, USA).
